# The Influence of the Nervous System upon Pathological Changes in the Skin

**Published:** 1882-06

**Authors:** 


### THE INFLUENCE OF THE NERVOUS SYSTEM UPON PATHOLOGICAL CHANGES IN THE SKIN.

Irsai (*Neurologisches Centralblatt*, No. 6, 1882), arrives at the following conclusions from his experiments:

1. Unilateral injuries of the spinal cord, or the injection of irritant substances into the spinal marrow, are followed by a corresponding paralysis, but not by any disease of the skin.

2. Several days after the infliction of the injury, an eruption of herpes was observed on the injured side, which also appeared on the opposite side if the inflammatory action in the cord extended to the other side.

3. The eruption lasted but a short time.

4. The eruption was accompanied by atrophy of the skin over a corresponding area.

5. In the injured spinal cord sclerosis appeared six to ten weeks after the infliction of the injury. At the same time vacuole-formation was observed in the anterior horns of the injured side, above the seat of injury. This was found in all cases.

